# Short (2‐Hour) Non‐Oxygenated End‐Ischemic Hypothermic Perfusion Versus Cold Storage in the Setting of Renal Transplantation

**DOI:** 10.1111/aor.14953

**Published:** 2025-01-24

**Authors:** Franco Ruberto, Quirino Lai, Mario Piazzolla, Luca Poli, Veronica Zullino, Giulia Diamantini, Matteo Brisciani, Francesco Giovanardi, Fabio Melandro, Silvia Quaresima, Massimo Rossi, Manuela Garofalo, Francesco Pugliese

**Affiliations:** ^1^ Department of Anesthesiology, Critical Care Medicine and Pain Therapy Sapienza University of Rome Rome Italy; ^2^ General Surgery and Organ Transplantation Unit Sapienza University of Rome Rome Italy

**Keywords:** deceased brain death, delayed graft function, Karpinski score, propensity score, urine output

## Abstract

**Background:**

Kidney transplantation (KT) is the most effective treatment for end‐stage renal disease. End‐ischemic hypothermic machine perfusion (EI‐HMP) has emerged as a promising method for preserving grafts before transplantation. This study aimed to compare graft function recovery in KT recipients of deceased brain‐death (DBD) grafts preserved with EI‐HMP versus static cold storage (SCS). The primary outcome was the rate of delayed graft function (DGF). Secondary outcomes included urine output, intensive care unit (ICU) stay, hospital stay duration, and survival rates.

**Methods:**

A retrospective, single‐center observational study was conducted at Sapienza University of Rome, analyzing 313 KT patients between January 2014 and September 2021. Patients were stratified into two groups based on graft preservation methods (EI‐HMP, *n* = 95; SCS, *n* = 218). A stabilized inverse probability treatment weighting (IPTW) method was employed to adjust for potential confounders.

**Results:**

There were no significant differences in DGF rates between the two groups (17.9% vs. 15.6% in SCS and EI‐HMP cases, respectively; *p* = 0.75). EI‐HMP group demonstrated a higher urine output on day 2 (*p* = 0.046), a shorter ICU stay (*p* < 0.0001), and a trend toward a shorter overall hospital stay (*p* = 0.07). No statistically significant differences were found between EI‐HMP and SCS cases in 1‐ and 3‐year overall survival rates (3.2% and 6.7% vs. 5.6% and 6.6%, respectively; log‐rank *p* = 0.53) or in death‐censored graft loss rates (5.4% and 8.9% vs. 5.7% and 7.3%, respectively; log‐rank *p* = 0.88). In a sub‐analysis of expanded criteria donors (ECD), EI‐HMP demonstrated a protective effect by reducing the risk of DGF (OR = 0.31, 95% CI = 0.09–0.95; *p* = 0.047).

**Conclusion:**

EI‐HMP was associated with certain short‐term benefits, including increased urine output and reduced ICU stays, but showed no significant impact on long‐term survival outcomes. A reduction in DGF rates was observed only in the ECD subgroup. Randomized controlled trials are necessary to further investigate the long‐term clinical benefits of EI‐HMP.

## Graphical abstract

EI‐HMP enhances urine output and reduces ICU stay in kidney transplant patients, particularly in ECD grafts, but does not significantly impact long‐term survival or dialysis avoidance. These short‐term benefits suggest EI‐HMP may improve early recovery, although its long‐term efficacy requires further investigation through randomized controlled trials.

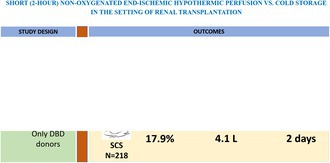

**Abbreviations**
BMI: Body Mass Index
CI: confidence interval
CIT: cold ischemia time
CMV‐Ab: cytomegalovirus antibody
CRR7: creatinine reduction ratio at day 7
CVA: cerebrovascular accident
DBD: deceased brain death
DCD: donors after cardiac death
DGF: delayed graft function
ECD: expanded criteria donors
EI‐HMP: end‐ischemic hypothermic machine perfusion
GS: graft survival
HbS‐Ab: hepatitis B surface antibody
HCV‐Ab: hepatitis C virus antibody
HLA: human leukocyte antigen
ICU: intensive care unit
IPTW: inverse probability therapy weighting
KDPI: Kidney Donor Profile Index
KDRI: Kidney Donor Risk Index
KT: kidney transplantation
OR: odds ratio
OS: overall survival
PRA: panel reactive antibody
SCS: static cold storage
SPSS: statistical package for the social sciences
STROBE: strengthening the reporting of observational studies in epidemiology
T2DM: type‐2 diabetes mellitus
WIT: warm ischemia time

## 1 Introduction

Kidney transplantation (KT) is widely regarded as the gold‐standard treatment for end‐stage renal disease [1]. However, its remarkable success, characterized by excellent post‐transplant survival rates, has led to a growing disparity between the supply of and demand for transplantable grafts [2]. To address this gap, the transplant community has increasingly turned to the use of non‐standard kidney grafts, including those from expanded criteria donors (ECDs) and donors after cardiac death (DCD) [3, 4]. Unfortunately, these grafts are often associated with poorer clinical outcomes [5].

To mitigate these challenges, hypothermic machine perfusion (HMP) has been widely adopted as a strategy to improve clinical outcomes by optimizing the preservation of marginal grafts [6]. The increasing application of HMP has also shifted paradigms in kidney preservation, suggesting that its benefits may extend beyond marginal grafts to standard kidney grafts, potentially influencing the entire post‐KT course [7].

The optimal timing for initiating perfusion—whether immediately after organ procurement or following a period of cold storage—remains a topic of ongoing debate. Limited evidence is available regarding the efficacy of end‐ischemic (EI)‐HMP in this context [8].

Based on these considerations, we hypothesized that post‐KT graft function recovery would be faster in patients receiving deceased brain death (DBD) single grafts treated with EI‐HMP compared to those preserved with static cold storage (SCS). At our center, we have adopted a non‐oxygenated EI‐HMP protocol for organ recovery, ensuring at least 2 h of perfusion before implantation.

In this study, we compared the post‐KT clinical outcomes of two preliminarily balanced groups of patients who underwent KT with DBD grafts, one group treated with non‐oxygenated EI‐HMP and the other preserved with SCS.

## 2 Materials and Methods

### 2.1 Study Design

This is a retrospective monocenter observational study investigating the data of patients undergoing a KT using a DBD graft.

The study was approved by the Local Ethics Board of AOU Policlinico Umberto I di Roma (approval number 1000/2018). The Strengthening the Reporting of Observational Studies in Epidemiology (STROBE) guidelines were followed to create the study.

### 2.2 Setting

The involved center was the Sapienza University of Rome, AOU Policlinico Umberto I Hospital, Rome.

### 2.3 Population

Between January 2014 and September 2021, 313 adult patients (aged ≥ 18 years) underwent consecutive kidney transplants at Sapienza University of Rome. All grafts were sourced from DBDs, with no exclusion criteria applied. The study population was divided into two groups based on the method of graft preservation: the SCS Group (*n* = 218, 69.6%) and the EI‐HMP Group (*n* = 95, 30.4%).

### 2.4 Outcomes

The study's primary outcome was the recovery of the transplanted graft after a single KT, which was defined as the absence of a post‐transplant delayed graft function (DGF) calculated on day seven after KT. Secondary outcomes were: (a) urine outputs at days 2 and 7, (b) intensive care unit (ICU) stay duration, (c) hospital stay duration, (d) overall survival (OS), and (e) death‐censored graft survival (GS). The last follow‐up date was September 30, 2024.

### 2.5 Data Collection

Data were retrospectively carried out on the prospectively collected charts of the patients. The guarantor of the data quality was the Data Manager of the Study Group (QL). Data errors and missingness were identified across the database and solved, when possible, with specific queries.

### 2.6 Definitions

DGF was defined using two criteria: (a) need for dialysis within 7 days from KT [9]; and (b) creatinine reduction ratio at day 7 (CRR7) ≤ 70%. CRR7 was calculated using the following equation: CRR7(%) = ([sCr0–sCr7] × 100)/sCr0.

sCr0 corresponded to the sCr levels immediately before KT and no later than 6 h after the last dialysis; Cr7 corresponded to the sCr levels at post‐KT day 7 [10].

Cold ischemia time (CIT) was calculated from the cross‐clamp time until the end of the EI‐HMP. Warm ischemia time (WIT) was calculated from the removal of the kidney from EI‐HMP to reperfusion with warm blood, inclusive of surgical anastomosis time.

The quality of the grafts was calculated using the Kidney Donor Risk Index (KDRI) and the Kidney Donor Profile Index (KDPI). The last available scaling factor was used for calculating the KDPI (year = 2022; number = 1.32896241147372) [11].

An ECD was defined according to the following criteria: (a) donor age ≥ 60 years; or (b) donor age of 50–59 years with two or more of the following comorbidities: a history of hypertension, death resulting from cerebrovascular accident (CVA), and terminal sCr ≥ 1.5 mg/dL [10]. In all these cases, a pre‐KT biopsy of the graft was done with the intent to establish the transplantability of the organ.

A group of expert pathologists assessed the histological graft quality using the Karpinski Score [12]. In all the cases, the histological quality information was available before the beginning of the KT. Grafts presenting a Karpinski Score ≤ 3 were judged to be usable for a single KT. In case of a Score ranging from 4 to 6, we decided case by case to use the graft for a single KT according to the following parameters: (a) absence of diffuse gross vascular atherosclerosis in the graft; (b) absence of severe microscopically assessed pyelonephritis. In no case, we perform a double KT. When the Score was ≥ 7, the kidney was directly discarded.

Patient death was defined as any event of death reported in the cohort by any cause during the entire follow‐up period. Graft loss was defined as returning to dialysis or sCr clearance < 15 mL/min/1.73 m^2^ at the last evaluation. The estimation of graft survival was censored in the case of patient death with a functioning graft.

### 2.7 Organ Procurement and Perfusion Machine Procedure

During organ procurement, in vivo, perfusion of abdominal organs was carried out using Celsior solution (IGL ITALIA SRL, Teolo, Italy) at a volume of 1 L per 10 kg of donor weight.

At our center, all perfused kidneys underwent an in‐house, non‐oxygenated end‐ischemic hypothermic machine perfusion (EI‐HMP) protocol following back‐table surgery. The LifePort Kidney Transporter machine (Organ Recovery Systems, Brussels, Belgium) was used to perform the perfusion, utilizing a pulsatile pressure mode. The initial perfusion pressure was set at 30 mmHg. KPS‐1 solution (Organ Recovery Systems, Brussels, Belgium) was employed as the perfusion medium, with its temperature maintained between 1°C and 8°C throughout the procedure.

Key parameters, including renal resistance (mmHg/mL/min), flow (mL/min), and temperature (°C), were recorded at specific time points: at the beginning of perfusion, 20, 40, 60, and 120 min. Each graft was perfused for a minimum of 120 min.

Between January 2014 and October 2017, hypothermic machine perfusion was not available in our center, and 146 consecutive kidney transplants utilized SCS preservation. In November 2017, the clinical practice was enhanced with the introduction of two perfusion machines. From November 2017 to September 2021, 167 kidney transplants were performed, of which 72 used SCS preservation and 95 utilized EI‐HMP.

The decision to use EI‐HMP for graft was made on a case‐by‐case basis, guided by the following considerations: (a) The use of an ECD graft; (b) prediction of a prolonged CIT exceeding 12 h; (c) recipient logistics, such as the need for dialysis before KT or significant distance between the recipient's residence and the transplant hospital, potentially delaying the initiation of KT.

### 2.8 Immunosuppression

Induction was performed using thymoglobulin (1.5 mg/kg/day for 4 days) in highly sensitized recipients (panel reactive antibody [PRA] ≥ 90%) and basiliximab (20 mg i.v. on day 0 and day 4) in the other patients. In all the patients, the initial maintenance therapy was based on tacrolimus (target trough levels of 8–12 ng/mL for the first 3 months), mycophenolate mofetil (1 g twice daily) or mycophenolate sodium (720 mg twice daily), and steroids with rapid taper.

### 2.9 Statistical Analysis

Continuous variables were reported as medians and 1st–3rd quartiles (Q1–3). Categorical variables were described as numbers and percentages. Comparisons between groups were made using Fisher's exact or chi‐square tests for categorical variables, as appropriate. Mann–Whitney was used for continuous variables. Missing data relative to the study covariate involved less than 10% of patients. Missing data were reported in detail in Table S1. In all the cases, missing data were handled with a single imputation method. In detail, a median of nearby points imputation was adopted. The median instead of the mean was adopted due to the skewed distribution of the managed variables.

The entire population was divided into two groups according to the use of EI‐HMP.

With the intent to compensate for the non‐randomized design of this retrospective study, the two groups were “balanced” using a stabilized inverse probability therapy weighting (IPTW).

A propensity score for each patient in the original population was generated. The score was created using a multivariate logistic regression model considering the need for dialysis within the first week after KT (no vs. yes) as the dependent variable. We selected 30 possible clinically relevant confounders as covariates: patient age, patient male sex, patient Body Mass Index (BMI), retransplantation, pre‐emptive KT, dialysis duration, patient arterial hypertension, patient type‐2 diabetes mellitus (T2DM), patient cytomegalovirus‐antibody (CMV‐Ab) positivity, patient hepatitis B surface‐antibody (HbS‐Ab) positivity, patient hepatitis C virus‐antibody (HCV‐Ab) positivity, maximum level of PRA before transplant, donor age, donor male sex, donor BMI, donor CMV‐Ab positive, CVA as the cause of donor death, donor hypotension(s), donor cardiac arrest(s), donor history of arterial hypertension, donor history of T2DM, ECD, surgical complexity caused by multiple anastomoses, donor serum creatinine (sCr), donor ICU stay, KDRI, KDPI, human leukocyte antigen (HLA) mismatch, CIT, and WIT.

All the covariates were available before the end of the KT procedure to avoid the risk of a possible immortal time bias in covariate selection.

With the intent to reduce the artificial modification of the sample size in the pseudo data, we used stabilized weights (SW) according to the formula:

$$\begin{array}{c} \mathrm{SW}=p/\mathrm{PS}\;\text{for the study group},\text{and}\\ \mathrm{SW}=\left(1-p\right)/\left(1-\mathrm{PS}\right)\;\text{for the control group} \end{array}$$

where *p* is the probability of etiology without considering covariates, and PS is the propensity score.

Because *p*‐values can be biased from population size, results from the comparisons between covariates subgroups were reported as effect size (Cohen's *D* value): values lower than |0.1| indicated very small differences between means, values between |0.1| and |0.3| indicated small differences, values between |0.3| and |0.5| indicated moderate differences, and values greater than |0.5| indicated considerable differences.

Multivariable logistic regression analyses were run after the stabilized IPTW to identify the risk factors for DGF after KT. According to previous research papers focused on the same argument, the investigated variables were selected using a “full model” approach. A backward Wald method was finally adopted for constructing the final model. Odds ratios (OR) and 95.0% confidence intervals (CI) were reported for significant variables.

OS and death‐censored GS probabilities were estimated using the Kaplan–Meier method. The survival results were compared using the log‐rank test.

Variables with a *p* < 0.05 were considered statistically significant. Statistical analyses and plots were run using the SPSS statistical package version 27.0 (SPSS Inc., Chicago, IL, USA).

## 3 Results

### 3.1 Study Population

Between January 2014 and September 2021, 313 KT patients meeting the inclusion criteria of this study were transplanted. The pre‐KT characteristics of the studied population are summarized in Table 1. Only minimal differences were observed between the SCS and EI‐HMP Groups. For example, KT recipients in both groups were similar in terms of age, sex, underlying renal disease, retransplantation rates, and history of arterial hypertension or T2DM. Differences were noted in BMI and dialysis duration, with the EI‐HMP group having higher BMI values and shorter dialysis durations.

**TABLE 1:** Pre‐KT characteristics of the investigated population divided in the two groups.

Variables	SCS (*n* = 218, 69.6%)	EI‐HMP (*n* = 95, 30.4%)	*p*
	Median (Q1–3) or *n* (%)		
Patient			
Age, years	53 (44–61)	53 (45–62)	0.52
Male sex	130 (59.6)	51 (53.7)	0.38
Caucasian	201 (92.2)	91 (95.8)	0.33
Height, cm	168 (163–175)	167 (160–172)	0.10
Weight, kg	70 (61–77)	72 (63–79)	0.16
BMI	24.2 (22.1–26.4)	25.4 (22.8–28.1)	0.004
Underlying renal disease			
ADPKD	40 (18.3)	18 (18.9)	0.88
GN	97 (44.5)	43 (45.3)	0.90
PN	5 (2.3)	3 83.2)	0.70
Nephroangiosclerosis	31 (14.2)	15 (15.8)	0.73
VUR	9 (4.1)	4 (4.2)	1.00
Unknown	15 (6.9)	2 (2.1)	0.11
Other	21 (9.6)	10 (10.5)	0.84
Retransplantation	31 (14.2)	12812.6)	0.86
Pre‐emptive KT	11 (5.0)	3 (3.2)	0.56
Dialysis duration, years	3 (2–6)	3 (1–4)	0.004
Arterial hypertension	181 (83.0)	81 (85.3)	0.74
T2DM	21 (9.6)	11 (11.6)	0.69
CMV‐Ab positive	204 (93.6)	84 (88.4)	0.17
HbS‐Ab positive	85 (39.0)	32 (33.7)	0.45
HCV‐Ab positive	6 (2.8)	6 (6.3)	0.20
Max PRA level before transplant	0 (0–0)	0 (0–0)	0.43
Donor			
Age, years	55 (43–66)	55 (47–65)	0.34
≥ 65 years	65 (29.8)	25 (26.3)	0.59
Male sex	105848.2)	50852.6)	0.54
Height, cm	170 (164–175)	170 (165–175)	0.23
Weight, kg	75 (66–86)	75 (72–83)	0.72
BMI	26.1 (24.2–28.9)	26.0 (24.6–27.8)	0.47
CMV‐Ab positive	192888.1)	87 (91.6)	0.43
HCV‐Ab positive	0 (−)	1 (1.1)	0.30
Cause of death			
CVA	160 (73.4)	73 (76.8)	0.58
Anoxia	14 (6.4)	5 (5.3)	0.80
Trauma	38 (17.4)	17 (17.9)	1.00
Other	6 (2.8)	0 (−)	0.18
Hypotension episode(s)	35 (16.1)	17 (17.9)	0.74
Cardiac arrest episode(s)	26 (11.9)	8 (8.4)	0.43
Arterial hypertension	63 (28.9)	27 (28.4)	1.00
T2DM	12 (5.5)	13 (13.7)	0.02
ECD	91 (41.7)	42 (44.2)	0.71
Surgical complexity	14 (6.4)	12 (12.6)	0.08
sCr, mg/dL	0.9 (0.6–1.1)	0.9 (0.8–1.1)	0.08
ICU stay, stay	4 (3–6)	4 (2–5)	0.17
KDRI	1.46 (1.13–2.05)	1.50 (1.25–2.05)	0.27
KDPI	1.11 (0.85–1.54)	1.13 (0.94–1.54)	0.27
Transplantation			
HLA mismatch	3 (3–4)	3 (3–4)	0.50
CIT, min	715 (600–870)	717 (610–868)	0.85
< 12 h	124 (56.9)	47 (49.5)	
12–24 h	93 (42.7)	47 (49.5)	0.42
> 24 h	1 (0.5)	1 (1.1)	
WIT, min	53 (45–60)	50 (40–55)	0.14
Induction with thymoglobulin	13 (6.0)	3 (3.2)	0.41

Abbreviations: Ab, antibody; ADPKD, autosomal dominant polycystic kidney disease; BMI, Body Mass Index; CIT, cold ischemia time; CMV, cytomegalovirus; CVA, cerebrovascular accident; ECD, expanded criteria donor; EI‐HMP, end‐ischemic hypothermic machine perfusion; GN, glomerulonephritis; HbS, hepatitis B surface; HCV, hepatitis C virus; HLA, human leukocyte antigen; ICU, intensive care unit; KDPI, kidney donor profile index; KDRI, kidney donor risk index; KT, kidney transplantation; *n*, number; PN, pyelonephritis; PRA, panel reactive antibody; Q1–3, 1st–3rd quartile; sCr, serum creatinine; SCS, static cold storage; T2DM, type‐2 diabetes mellitus; VUR, vesicoureteral reflux; WIT, warm ischemia time.

Regarding donor characteristics, only the history of T2DM was significantly different, with higher rates in the EI‐HMP group. Donor age, sex, BMI, cause of death, hemodynamic instability, history of arterial hypertension, serum creatinine at the time of procurement, ICU stay, KDRI, KDPI, and ECD status were similar between the groups. Additionally, CIT and WIT at the time of KT were comparable between the two groups.

### 3.2 Perfusion Decision

The decision to perfuse a graft was not randomized but made on a case‐by‐case basis. Specifically, graft perfusion was considered in 42/95 (44.2%) cases due to ECD grafts, in 43 (45.3%) cases due to predicted long CIT (> 12 h), and in 10 (10.5%) cases due to recipient logistics (e.g., dialysis needs before KT or long distance from transplant center).

### 3.3 Stabilized Inverse Probability of Treatment Weighting (IPTW) Adjustment

Despite some initial differences between the two groups, an artificial balance was achieved using stabilized IPTW to minimize potential biases in this non‐randomized, retrospective study. As shown in Table 2, IPTW balancing effectively adjusted for 30 potential confounders. Before IPTW, 10 variables showed very small differences, 19 showed small differences, and 1 showed moderate differences. After IPTW, 27 variables showed very small differences and only 3 showed small differences. The IPTW adjustment did not alter the sample size in the pseudo‐population.

**TABLE 2:** Effect of stabilized IPTW in the population on the variables used for balancing the two groups.

Variables	Pre‐IPTW			Post‐IPTW		
	SCS (*n* = 218)	EI‐HMP (*n* = 95)	Cohen's *D*‐value	SCS (*n* = 218)	EI‐HMP (*n* = 95)	Cohen's *D*‐value
	Mean (±SD)			Mean (±SD)		
Patient age	51.71 ± 12.21	53.12 ± 12.12	0.05	51.94 ± 12.43	51.52 ± 11.88	0.05
Patient male sex	0.59 ± 0.49	0.54 ± 0.50	−0.12	0.59 ± 0.49	0.60 ± 0.49	0.03
Patient BMI	24.31 ± 3.42	25.67 ± 3.95	0.10	24.64 ± 3.43	24.59 ± 3.72	−0.02
Retransplantation	0.14 ± 0.35	0.13 ± 0.34	−0.36	0.14 ± 0.34	0.13 ± 0.33	0.01
Pre‐emptive KT	0.05 ± 0.22	0.03 ± 0.17	0.04	0.04 ± 0.21	0.03 ± 0.18	0.03
Dialysis duration	4.73 ± 4.02	3.70 ± 3.33	0.12	4.40 ± 3.74	4.97 ± 4.30	0.06
Patient arterial hypertension	0.83 ± 0.38	0.86 ± 0.35	0.29	0.84 ± 0.37	0.86 ± 0.34	−0.14
Patient T2DM	0.09 ± 0.29	0.12 ± 0.33	−0.10	0.10 ± 0.30	0.09 ± 0.28	−0.06
Patient CMV‐Ab positive	0.94 ± 0.24	0.88 ± 0.33	−0.08	0.92 ± 0.27	0.92 ± 0.27	0.04
Patient HbS‐Ab positive	0.38 ± 0.49	0.37 ± 0.48	0.19	0.38 ± 0.49	0.42 ± 0.50	−0.01
Patient HCV‐Ab positive	0.03 ± 0.17	0.06 ± 0.24	0.02	0.04 ± 0.20	0.04 ± 0.19	−0.09
Max PRA before transplant	11.54 ± 28.11	14.33 ± 31.06	−0.09	11.73 ± 28.05	14.58 ± 36.58	−0.08
Donor age	52.89 ± 16.38	55.87 ± 15.73	−0.14	53.57 ± 16.53	53.29 ± 16.25	0.02
Donor male sex	0.49 ± 0.50	0.51 ± 0.50	−0.19	0.49 ± 0.50	0.53 ± 0.50	0.02
Donor BMI	26.63 ± 3.70	26.36 ± 3.35	−0.06	26.51 ± 3.70	26.32 ± 3.41	−0.08
Donor CMV‐Ab positive	0.88 ± 0.33	0.92 ± 0.27	0.08	0.89 ± 0.31	0.90 ± 0.30	0.05
CVA as donor cause of death	0.73 ± 0.45	0.78 ± 0.41	−0.15	0.74 ± 0.44	0.71 ± 0.46	−0.04
Donor hypotension(s)	0.16 ± 0.37	0.18 ± 0.38	−0.13	0.16 ± 0.36	0.14 ± 0.35	0.06
Donor cardiac arrest(s)	0.12 ± 0.33	0.08 ± 0.27	−0.05	0.11 ± 0.31	0.11 ± 0.32	0.04
Donor arterial hypertension	0.30 ± 0.46	0.27 ± 0.44	0.15	0.30 ± 0.46	0.31 ± 0.47	−0.01
Donor T2DM	0.06 ± 0.23	0.13 ± 0.34	0.07	0.08 ± 0.28	0.08 ± 0.27	−0.03
ECD	0.41 ± 0.49	0.47 ± 0.50	−0.24	0.43 ± 0.50	0.43 ± 0.50	0.01
Surgical complexity	0.06 ± 0.24	0.13 ± 0.34	−0.12	0.08 ± 0.28	0.08 ± 0.27	0.00
Donor sCr	1.00 ± 0.72	1.10 ± 0.72	−0.22	1.02 ± 0.80	1.02 ± 0.57	0.02
Donor ICU stay	5.44 ± 5.85	4.71 ± 4.26	−0.14	5.24 ± 5.23	4.75 ± 3.97	0.00
KDRI	1.56 ± 0.55	1.67 ± 0.66	−0.17	1.59 ± 0.56	1.57 ± 0.63	0.03
KDPI	1.21 ± 0.43	1.30 ± 0.50	0.15	1.24 ± 0.44	1.23 ± 0.47	0.11
HLA mismatch	3.33 ± 1.03	3.34 ± 0.92	−0.01	3.35 ± 1.04	3.34 ± 1.00	0.01
CIT	723.91 ± 202.41	742.25 ± 219.74	−0.18	728.90 ± 196.49	730.09 ± 242.69	0.02
WIT	53.02 ± 17.23	49.54 ± 13.17	0.24	51.50 ± 14.52	50.03 ± 13.51	0.11

Abbreviations: Ab, antibody; BMI, Body Mass Index; CIT, cold ischemia time; CMV, cytomegalovirus; CVA, cerebrovascular accident; ECD, expanded criteria donor; EI‐HMP, end‐ischemic hypothermic machine perfusion; HbS, hepatitis B surface; HCV, hepatitis C virus; HLA, human leukocyte antigen; ICU, intensive care unit; IPTW, inverse probability therapy weighting; KDPI, kidney donor profile index; KDRI, kidney donor risk index; KT, kidney transplantation; *n*, number; PRA, panel reactive antibody; sCr, serum creatinine; SCS, static cold storage; SD, standard deviation; T2DM, type‐2 diabetes mellitus; WIT, warm ischemia time.

### 3.4 Post‐KT Clinical Course in the Balanced Groups

**FIGURE 1:**
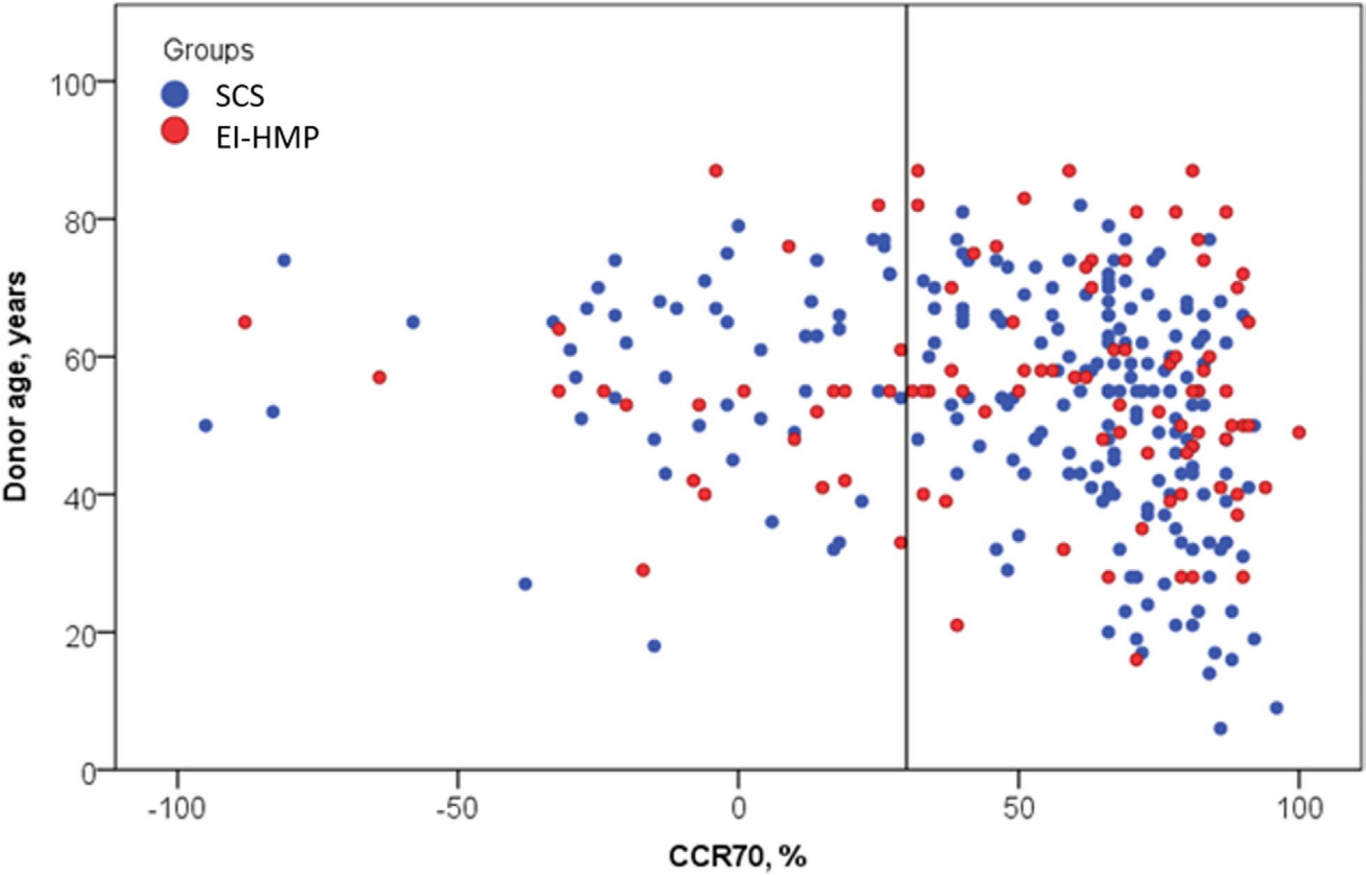
Distribution of SCS and EI‐HMP cases according to donor age and modification of serum creatinine at day 7 after transplantation. [Color figure can be viewed at wileyonlinelibrary.com]

The post‐transplant clinical outcomes after IPTW adjustment are summarized in Table 3. No significant differences were found between the groups regarding post‐KT DGF rates. Specifically, dialysis within the first week after KT was required in 17.9% of SCS cases and 15.6% of EI‐HMP cases (*p* = 0.75). Similarly, a creatinine clearance ratio on day 7 (CCR7) < 70% was observed in 22.9% of SCS cases versus 19.8% of EI‐HMP cases (*p* = 0.66) (Figure 1).

**TABLE 3:** Post‐transplant clinical course observed in the two groups (pseudo populations after IPTW balancing) in the entire population and in the sub‐population of patients receiving ECD grafts.

Variables	SCS (*n* = 218)	EI‐HMP (*n* = 95)	*p*
	Median (Q1–3) or *n* (%)		
*Entire population*			
DGF (need for dialysis)	39 (17.9)	15 (15.6)	0.75
DGF (CCR7 < 70%)	50 (22.9)	19 (19.8)	0.66
CCR7	66 (35–76)	66 (35–82)	0.12
Urine output at day 2, mL	4100 (2401–5502)	4193 (3500–6215)	0.046
Urine output at day 7, mL	3500 (3000–4543)	3500 (2628–4000)	0.03
ICU stay, days	2 (1–3)	1 (1–2)	< 0.0001
Hospital stay, days	12 (10–18)	11 (9–15)	0.07

Variables	SCS (*n* = 92)	EI‐HMP (*n* = 39)	*p*
	Median (Q1–3) or *n* (%)		
*ECD*			
DGF (need for dialysis)	22 (23.9)	3 (7.7)	0.03
DGF (CCR7 < 70%)	30 (32.6)	8 (20.5)	0.21
CCR7	53 (18–66)	62 (36–79)	0.07
Urine output at day 2, mL	4100 (1620–4776)	4100 (3127–6301)	0.10
Urine output at day 7, mL	3500 (3000–4430)	3370 (2650–3800)	0.15
ICU stay, days	2 (1–3)	1 (1–2)	0.002
Hospital stay, days	12 (11–21)	11 (8–14)	0.007

Abbreviations: CCR7, creatinine reduction ratio at day 7; DGF, delayed graft function; ECD, expanded criteria donors; EI‐HMP, end‐ischemic hypothermic machine perfusion; ICU, intensive care unit; *n*, number; Q1–3, 1st–3rd quartile; SCS, static cold storage.

However, urine output differed significantly between the two groups, with higher values in the EI‐HMP group on day 2 (*p* = 0.046) and lower values on day 7 (*p* = 0.003). The length of stay in the ICU was significantly shorter in the EI‐HMP group (*p* < 0.0001), and although the overall hospital stay was shorter in the EI‐HMP group, the difference was not statistically significant (*p* = 0.07).

### 3.5 Risk Factors for DGF

Multivariable logistic regression analyses identified the following risk factors for DGF in the post‐IPTW population (Table 4): When DGF was defined as the need for dialysis within the first 7 days post‐KT, cerebrovascular accident (CVA) as the cause of donor death (OR = 2.317, *p* = 0.04) was the only significant factor. Duration of recipient dialysis before KT approached statistical relevance (OR = 1.067, *p* = 0.07).

**TABLE 4:** Multivariable logistic regression analysis for the risk factors of DGF: (a) need for dialysis within the first post‐KT week; (b) creatinine reduction ratio at day 7 (CRR7) ≤ 70%.

Variables	Beta	SE	Wald	OR	95% CI	*p*
**(a) Need for dialysis within the first post‐KT week**						
Entire population*						
CVA as cause of donor death	0.840	0.400	4.423	2.317	1.059–5.071	0.04
Duration of dialysis before KT in years	0.065	0.036	3.290	1.067	0.99–1.059	0.07
ECD**						
EI‐HMP	−1.176	0.623	3.564	0.308	0.091–0.946	0.047
Duration of dialysis before KT in years	0.104	0.057	3.295	1.109	0.992–1.241	0.07
−2LogLikelihoods: 280.67 (*); 121.80 (**)						
**(b) Creatinine reduction ratio at day 7 (CRR7) ≤ 70%**						
Entire population*						
Recipient BMI	0.098	0.040	6.119	1.103	1.021–1.193	0.01
CVA as cause of donor death	0.785	0.376	4.369	2.193	1.050–4.580	0.04
ECD	0.555	0.286	3.775	1.742	0.995–3.050	0.052
ECD**						
Recipient BMI	0.135	0.057	5.579	1.144	1.023–1.280	0.02
−2LogLikelihoods: 312.10 (*); 153.15 (**)						

*Note:* Pseudo populations analyzed after IPTW balancing. Backward Wald method adopted for the construction of the models. Variables initially introduced into the mathematical models: patient age, patient BMI, retransplantation, preemptive transplantation, duration of dialysis, donor age, donor BMI, CVA as donor cause of death, anoxia as donor cause of death, donor cardiac arrest(s), donor history of hypertension, donor history of diabetes, ECD, surgical complexity due to multiple anastomoses, CIT, EI‐HMP use.

Abbreviations: BMI, Body Mass Index; CI, confidence intervals; CIT, cold ischemia time; CVA, cerebrovascular accident; ECD, expanded criteria donors; EI‐HMP, end‐ischemic hypothermic machine perfusion; KT, kidney transplantation; OR, odds ratio; SE, standard error.

When DGF was defined by CRR7 ≤ 70%, recipient BMI (OR = 1.103, *p* = 0.01) and CVA as the cause of donor death (OR = 2.193, *p* = 0.04) were significant risk factors. The use of ECD grafts (OR = 1.742, *p* = 0.052) was almost statistically relevant as a DGF risk factor.

### 3.6 Survival Curves

**FIGURE 2:**
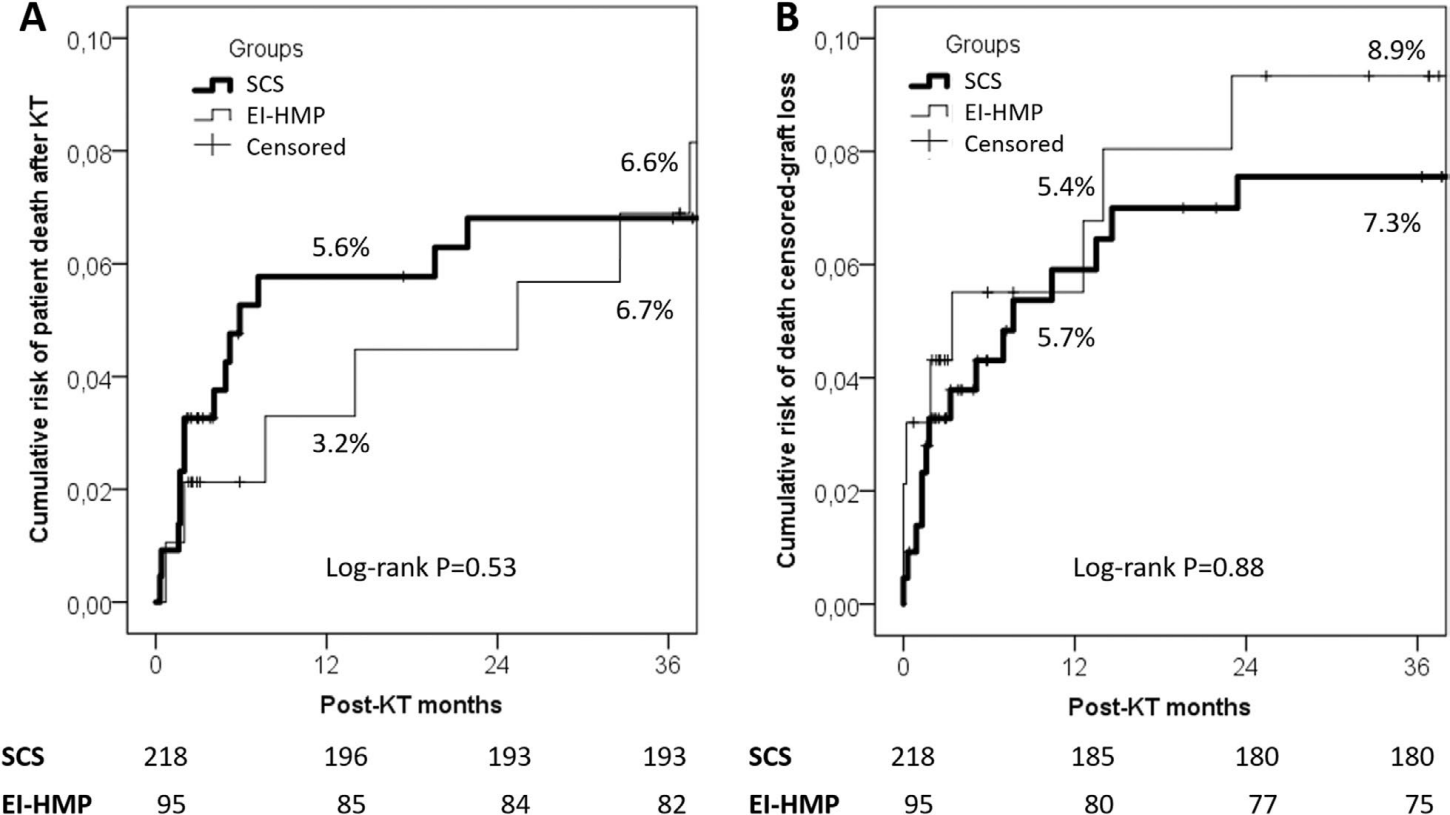
(A) Three‐year cumulative risk of patient death after KT in EI‐HMP and SCS groups; (B) three‐year cumulative risk of death‐censored graft loss after KT in EI‐HMP and SCS groups.

There were no significant differences in the cumulative patient death rates between the EI‐HMP and SCS groups. The 1‐ and 3‐year mortality rates were 3.2% and 6.7% in the EI‐HMP group versus 5.6% and 6.6% in the SCS group (log‐rank *p* = 0.53) (Figure 2A). Similarly, there were no significant differences in death‐censored graft loss rates. The 1‐ and 3‐year graft loss rates were 5.4% and 8.9% in the EI‐HMP group versus 5.7% and 7.3% in the SCS group (log‐rank *p* = 0.88) (Figure 2B).

### 3.7 Sub‐Analysis in ECD Graft Recipients

In the subgroup of patients receiving ECD grafts, significant differences were observed in the post‐KT clinical course when EI‐HMP was used. The need for dialysis within the first‐week post‐KT was significantly more frequent in the SCS group (23.9% vs. 7.7%; *p* = 0.03). The median creatinine reduction at day 7 was higher in the EI‐HMP group (62% vs. 53%; *p* = 0.07), although not statistically significant. ICU and overall hospital stays were consistently shorter in the EI‐HMP group (*p* = 0.002 and *p* = 0.007, respectively).

Exploring risk factors for DGF in ECDs, the use of EI‐HMP was protective against DGF (OR = 0.308, *p* = 0.047). In contrast, the recipient's dialysis duration was a relevant risk factor (OR = 1.109, *p* = 0.07). For CRR7 ≤ 70%, recipient BMI (OR = 1.144, *p* = 0.02) was the only independent risk factor.

Regarding survival rates in ECD graft recipients, there were no significant differences between EI‐HMP and SCS groups. The 1‐ and 3‐year patient mortality rates were 4.8% and 7.5% in the EI‐HMP group versus 11.3% and 12.5% in the SCS group (log‐rank *p* = 0.88). Likewise, there were no significant differences in death‐censored graft loss rates, with 1‐ and 3‐year rates of 7.2% and 8.5% in the EI‐HMP group versus 7.1% and 12.9% in the SCS group (log‐rank *p* = 0.67).

## 4 Discussion

Based on the findings of this study, a potential beneficial role of EI‐HMP should be postulated in terms of increased urine output and a shorter ICU stay. These differences may indicate advantages in the recovery phase or fewer immediate post‐transplant complications with EI‐HMP. Notably, EI‐HMP was also identified as a protective factor against DGF in ECD patients, suggesting that EI‐HMP might mitigate some risks related to pre‐transplant graft quality. However, these advantages do not translate into significant differences in dialysis avoidance or long‐term patient survival and graft loss rates.

A significant strength of the present study is that the effects correlated with EI‐HMP were investigated using a “balanced” design, which minimized potential biases inherent in this study's non‐randomized, retrospective nature through the IPTW approach. To our knowledge, this is the first time such a statistical method has been adopted to investigate the EI‐HMP approach in KT.

The clinical use of HMP is supported by over 15 years of experience, during which this strategy has been increasingly adopted to improve the recovery of marginal grafts [13]. However, the end‐ischemic approach has been largely explored in the liver transplant setting [14], with limited experiences reported in the clinical KT setting, mainly regarding long‐term survival rates [8].

A recent Cochrane meta‐analysis on various perfusion strategies (continuous vs. end‐ischemic) highlighted some differences among these approaches [8]. The majority of literature compares non‐oxygenated HMP with standard SCS, reporting a beneficial effect of HMP in reducing the DGF rate (relative risk [RR] = 0.78, 95% CI = 0.69–0.88; *p*‐value < 0.0001). Subgroup analysis comparing continuous HMP with SCS showed similar benefits (RR = 0.78, 95% CI = 0.64–0.96; *p*‐value < 0.0001) and an added protective effect on graft survival (hazard ratio [HR] = 0.46, 95% CI = 0.29–0.75; *p*‐value = 0.002).

In contrast, EI‐HMP did not show the same effects [8]. A recent RCT involving five European centers (*N* = 305) reported that oxygenated EI‐HMP may make little or no difference compared with SCS alone in the rate of DGF (30/127 EI‐HMP vs. 38/135 SCS; *p*‐value = 0.4), 12‐month graft survival (HR = 1.20, 95% CI = 0.49–2.96; *p*‐value = 0.69), incidence of acute rejection (23/127 EI‐HMP vs. 18/135 SCS; *p*‐value = 0.29), or mean glomerular filtration rate at 12 months (39.9 ± 14.4 vs. 41.2 ± 17.1; *p*‐value = 0.53) [15].

A meta‐analysis focusing solely on RCTs reaffirmed the limited efficacy of partial HMP in preventing DGF (RR = 0.92, 95% CI = 0.69–1.22) [16].

These findings align with our results, indicating that the end‐ischemic perfusion approach did not significantly impact dialysis avoidance or short‐term survival rates across the entire population.

Given these results, questions have been raised about the utility of EI‐HMP compared with continuous HMP, particularly concerning the additional costs without significant positive effects. Possible explanations for these, sometimes contradictory, results include insufficient statistical power in the partial HMP group [16].

Interestingly, our study found that perfusion played a statistically significant protective role in reducing the risk of dialysis during the first post‐transplant week (OR = 0.31, 95% CI = 0.09–0.95; *p*‐value = 0.047) in patients receiving ECD grafts. This finding is consistent with previous research indicating benefits from EI‐HMP for marginal deceased donor grafts. A previous study by our group reported that ECD grafts with a Karpinsky Score > 3 perfused with EI‐HMP had lower rates of DGF (8.7% vs. 34.4%; *p*‐value = 0.051), better reduction in serum creatinine (median at 7 days: 2.2 vs. 4.3 mg/dL; *p*‐value = 0.045), shorter hospital stays (median 11 vs. 15 days; *p*‐value = 0.01), and improved 3‐year death‐censored graft survival rates (91.3% vs. 77.0%) compared to SCS cases [6].

A study from The Netherlands, Belgium, and Germany demonstrated that the risk of DGF increased by 14% for each additional hour of CIT in ECD deceased donor grafts, compared to a 6% increase in non‐ECD deceased donor grafts and a 13% increase in DCD grafts. This datum underscores the vulnerability of these grafts and suggests that using a perfusion machine, even in the final hours of ischemic preservation, can correlate with some benefits [17].

Another significant strength of the present study is that we investigated the rapidity of urine output restoration following KT in EI‐HMP grafts, an intriguing but underexplored feature in previous research. The immediate restoration of urine output after transplant is likely related to the ability of machine perfusion to minimize ischemia–reperfusion injury, thereby reducing the long‐term clinical effects of acute tubular necrosis [18].

Several studies have found a correlation between urine output, DGF, and 1‐year graft function [19, 20]. Rapid recovery of urine output could be a key factor contributing to the shorter hospital stays observed in our study, impacting the duration of DGF. This effect not only has potential economic implications, balancing the higher costs of the perfusion machine against the reduced expenses for dialysis and hospital stays, but it may also enhance the overall availability of transplantable organs [21].

The study has some limitations that need to be addressed. Firstly, the study reports a retrospective non‐randomized series. Although an IPTW method was employed to mitigate the potential presence of biases, it is important to note this limitation. In fact, the absence of a randomized design in the study or the potential exclusion of biases from the IPTW calculation should be considered. Additionally, the study focused on the immediate post‐transplant benefits of EI‐HMP and did not explore long‐term outcomes. A more extended follow‐up period is necessary to determine whether the reported short‐term benefits translate into sustained clinical advantages. Furthermore, the study's findings show inconsistencies with previous research, indicating a need for further investigation to reconcile these differences. The possibility of insufficient statistical power in the partial HMP group is also acknowledged, which may contribute to the lack of significant differences observed in some outcomes.

In conclusion, some positive short‐term effects were observed using EI‐HMP, such as enhanced urine output and reduced ICU stays, but no significant impact was reported in terms of long‐term survival. DGF rate reduction was observed only in the sub‐group of ECDs. Randomized controlled trials are needed to explore the long‐term clinical benefits of EI‐HMP.

## Author Contributions

F.R. and Q.L. were responsible for the conception, design, analysis, and writing of the study. M.P., V.Z., G.D., M.B., F.G., F.M., S.Q., and M.G. were involved with the collection and interpretation of data. L.P., M.R., and F.P. participated in data management, review and editing of the manuscript.

## Conflicts of Interest

The authors declare no conflicts of interest.

## Supporting information


Table S1.
