# Supplementary material for: Short (2‐Hour) Non‐Oxygenated End‐Ischemic Hypothermic Perfusion Versus Cold Storage in the Setting of Renal Transplantation
Source: Artif Organs. 2025 Jan 24;49(5):831–41. doi: 10.1111/aor.14953 (PMC12019098; doi:10.1111/aor.14953)
Supplement: Supplementary file 1 — Table S1. [file AOR-49-831-s001.docx]

**Supplementary Table 1.** Missing data in the entire population (N=313).

| **Variables** | **Missing data (n)** | **(%)** |
| --- | --- | --- |
| **Patient** | | |
| **Age** | 0 | 0.0 |
| **Sex** | 0 | 0.0 |
| **Caucasian** | 0 | 0.0 |
| **Blood group** | 11 | 3.5 |
| **BMI** | 18 | 5.8 |
| **Renal disease** | 0 | 0.0 |
| **Re-KT** | 12 | 3.8 |
| **Pre-emptive** | 24 | 7.7 |
| **Years dyalisis** | 27 | 8.6 |
| **Arterial hypertension** | 22 | 7.0 |
| **T2DM** | 14 | 4.5 |
| **Donor** | | |
| **Age** | 0 | 0.0 |
| **Sex** | 0 | 0.0 |
| **Caucasian** | 0 | 0.0 |
| **Blood group** | 11 | 3.5 |
| **BMI** | 28 | 8.9 |
| **Cause of death** | 28 | 8.9 |
| **Arterial hypertension** | 30 | 9.6 |
| **T2DM** | 31 | 9.9 |
| **sCr** | 21 | 6.7 |
| **HCV status** | 0 | 0.0 |
| **Transplantation** | | |
| **CIT** | 25 | 8.0 |
| **Use of HMP** | 0 | 0.0 |
| **sCr time of KT** | 0 | 0.0 |
| **sCr time 7th post-KT day** | 0 | 0.0 |
| **Dialysis within the first week** | 0 | 0.0 |
| **Abbreviations:** BMI, body mass index; KT, kidney transplantation; T2DM, type-2 diabetes mellitus; sCr, serum creatinin; HCV, hepatitis C virus; HBV, hepatitis B virus; CIT, cold ischemia time; HMP, hypothermic machine perfusion. | | |
